# Global Workspace Theory (GWT) and Prefrontal Cortex: Recent Developments

**DOI:** 10.3389/fpsyg.2021.749868

**Published:** 2021-11-10

**Authors:** Bernard J. Baars, Natalie Geld, Robert Kozma

**Affiliations:** ^1^Center for the Future Mind, Florida Atlantic University, Boca Raton, FL, United States; ^2^MedNeuro, Inc., New York, NY, United States; ^3^Department of Mathematical Sciences, The University of Memphis, Memphis, TN, United States

**Keywords:** global workspace theory, prefrontal cortex (PFC), cortico-thalamic activity, conscious cognition, intracranial electrical stimulation, visual processing, metacognition, Neural Darwinism

## Introduction

In this work, we provide a brief overview of Global Workspace Theory (GWT), along with recent developments and clarifications of modern neuroscientific evidence. GWT started in the 1980s as a purely psychological theory of conscious cognition, and has become a prominent approach in scientific studies of consciousness (Mashour et al., 2020). Based on today's far more detailed understanding of the brain, GWT has adapted to new waves of evidence. The brain-based version of GWT is called Global Workspace Dynamics (GWD) (Baars et al., 2013; Baars and Geld, 2019) precisely because the cortex is viewed as a “unified oscillatory machine” (Steriade, 1999). GWT therefore joins other theories in viewing consciousness as the product of highly integrated and widespread cortico-thalamic (C-T) activity, following a long trail of evidence (Dehaene et al., 1998).

Here we aim to clarify some empirical questions that have been raised, and review evidence that the prefrontal and posterior regions support dynamic global workspace functions, in agreement with several other authors. Static, gross anatomical divisions are superseded by the dynamical connectome of cortex.

We aim to correct the following misunderstandings. In a recent paper, Raccah et al. (2021) claimed that the prefrontal cortex (PfC) is not causally involved in enabling consciousness, based on a review of intracranial electrical stimulation (iES) experiments. We will show that Raccah et al.'s claim that the prefrontal cortex (PfC) does not support consciousness is incorrect.

The brain evidence is now compelling that PfC indeed participates in the visual conscious stream, for example, and excellent evidence for that has emerged in recent years. We discuss the additional evidence and how that has a direct bearing on the PfC.

We also respond to Raccah et al.'s (2021) mistaken claim about the role of the prefrontal cortex and GWT. GWT does not assert that the prefrontal cortex (PfC) is essential for conscious vision, nor does it deny a role for the prefrontal lobe. The 1988 version of GWT made no assertions about the role of cortex in consciousness. These claims are mistaken, and indeed, self contradictory.

In addition, this integrated conception of cortex also answers counterclaims about consciousness and metacognition; therefore we address some misunderstandings about metacognition in the Global Workspace “family” of theories (Shea and Frith, 2019).

Shea and Frith (2019) proposed that “The Global Workspace Needs Metacognition.” However, in 1988 Baars already described two varieties of metacognition that are implied by GWT (Baars, 1988; Baars and Geld, 2019).

Here we have three objectives.

1. We provide a brief overview of Global Workspace Theory (GWT), and recent theoretical developments in light of modern evidence.
2. We address some of the claims in these two papers, following our previous brief response to the Raccah et al. publication (see also: https://www.jneurosci.org/content/41/10/2076.abstract#re-global-workspace-theory-gwt-and-prefrontal-cortex-a-reply-to-raccah-et-al).
3. We clarify some points related to the conscious brain, including metacognition. In particular, we make a fundamental distinction between CONSCIOUS metacognition and UN-conscious metacognition.

Cortex is extraordinarily flexible in its dynamic recruitment of different regions for different tasks. Therefore, an arbitrary division between prefrontal and other neuronal regions tends to be misleading. Consciousness requires a much broader, more integrative view (Bressler and Kelso, 2016).

## GWT and Cortex

### Global Workspace Theory (GWT): A Theory of Human Cognitive Architecture, the Cortex, and Consciousness. A Brief Overview

Far from being some free-floating cloud around our heads, sensory consciousness is profoundly embedded in biology, anatomy, physiology, and above all, in adaptive brain functions that serve us in every second of waking life. This is not some philosophical speculation. It is now supported by numerous empirical findings published in peer-reviewed journals that are easily found in web archives. How can we understand the evidence?

The best answer today is a “global workspace architecture,” first developed by cognitive modeling groups led by Alan Newell and Herbert A. Simon. The term “global workspace” comes from Artificial Intelligence, where it refers to a fleeting memory domain that allows for cooperative problem-solving by large collections of specialized programs. Some brain implications of the theory have been extensively explored over a 40 year period.

Global Workspace Theory (GWT) began with this question: “How does a serial, integrated and very limited stream of consciousness emerge from a nervous system that is mostly unconscious, distributed, parallel and of enormous capacity?” GWT is a widely used model for the role of conscious and unconscious events in the functioning of the brain, a set of explicit assumptions that can be tested, as many of them have been over several decades.

Global Workspace Dynamics (GWD) is the most current version of GWT—attempting to take into account the complexities of the living brain. Global Workspace Dynamics of conscious experience is supported by brain evidence, particularly the role of the cortex and thalamus. While the cortex and thalamus look separate to the naked eye, they act as an integrated system (Llinás and Paré, 1991; Edelman and Tononi, 2000; Steriade, 2006; Freeman, 2007).

Stanislas Dehaene and Jean-Pierre Changeux in Paris have developed experimentally testable models and made further testable claims about the brain basis of visual consciousness (Dehaene et al., 1998; Dehaene and Changeux, 2011). The Dehaene-Changeux Model (DCM) and Global Neuronal Workspace Theory (GNWT) are part of the larger GW “family” of related theories.

Stan Franklin and colleagues have built on GWT to sketch out a more general theory of cognition in LIDA, a computational implementation of GWT (Franklin et al., 2012).

These theoretical ideas provide a useful framework for our rapidly accumulating body of evidence. They are consistent with our current knowledge, and can be enriched to include other aspects of human experience. GWT-GWD neither denies nor asserts the dominance of any anatomical region; it is focused on the dynamic functioning of the cerebral cortex, and does not partition the cortex into static prefrontal and posterior divisions. Brain anatomy gives a necessary (but not sufficient) account of Global Workspace Dynamics.

### The GWT “Family” of Theories Is Evidence-Driven

The GW “family” of theories is essentially empirical. We believe that the following theories are in harmony with each other, including Dehaene et al. (1998), Edelman et al. (2011), Kozma and Freeman (2016), Tononi et al. (2016), Deco et al. (2019), and Mashour et al. (2020). Each approach is distinctive and each is based on a strong body of evidence; but they converge well.

Because GWT-GWD aims to account for an extensive body of evidence discussed by many authors, it is in accord with Edelman's Neural Darwinism, Freeman and Kozma's Neurodynamics, Tononi and Koch's Information Integration Theory, Dehaene and Changeux's Global Neuronal Workspace Theory, Mashour et al. and most recently, the functional connectomics of Deco, Vidaurre and Kringelbach.

### GWT and the Cortico-Thalamic System

A great deal of evidence supports the role of the cortico-thalamic (C-T) system in conscious experiences. GWT provides a reasonable interpretation of “ignitions” in the C-T system (Dehaene and Changeux, 2011). A dynamic conception of cortex suggests that PfC cannot be functionally separated from the rest of the C-T system. We show evidence that the prefrontal and posterior regions support dynamic global workspace functions, in agreement with several other authors (Edelman et al., 2011; Deco et al., 2019).

GW dynamics suggests that conscious experiences reflect a flexible “binding and broadcasting” function in the brain, which is able to mobilize a large, distributed collection of specialized cortical networks and processes that are not conscious by themselves. Note that the “broadcast” phase proposed by the theory should evoke widespread adaptation, for the same reason that a fire alarm should evoke widespread responding, because the specific needs for task-relevant responders cannot be completely known ahead of time. General alarms are interpreted according to local conditions.

A brain-based GW interacts with an “audience” of highly distributed, specialized knowledge sources, which interpret the global signal in terms of local knowledge (Baars, 1988). The global signal triggers reentrant signaling; resonance is the typical activity of the cortex.

**Figure 1:**
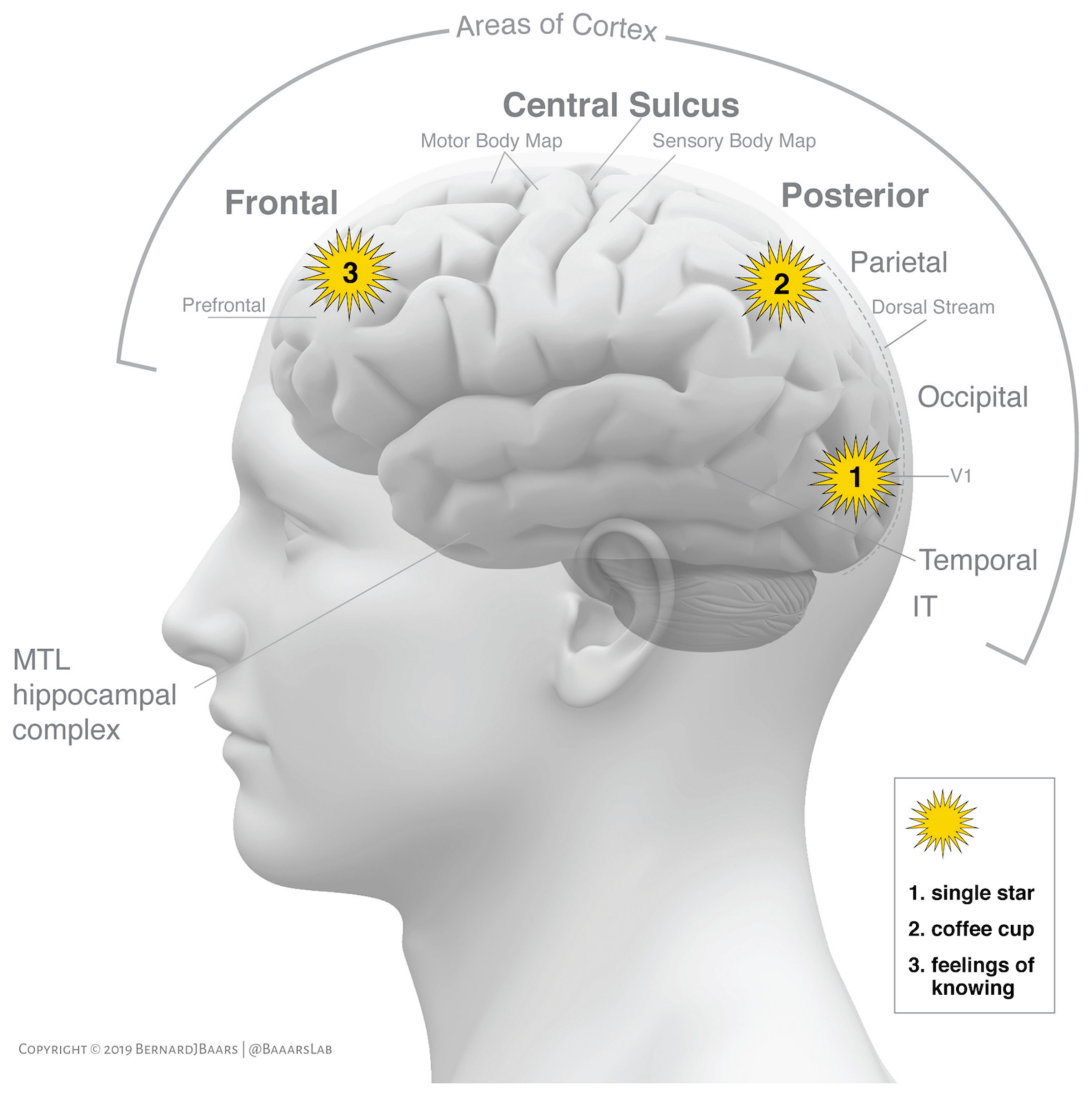
Shows three separate conscious events in a graphic interpretation of alternative sources of “ignition” in the C-T core (Baars and Geld, 2019, p. 581). All three involve binding and broadcasting (“ignition”) in three cortical locations. Area V1 is the first visual projection area in the cortex, and has the proper resolution and contrast representation to resolve the light from a single star on a dark night. Parietal cortex serves to contextualize conscious objects in egocentric and allocentric space, as in a coffee cup. The third “ignition” is hypothesized to emerge in the prefrontal cortex, plausibly involving a Feeling of Knowing (FOK), such as is induced by a tip-of-the-tongue task. (The hippocampus is not considered here, but it plays a major role in conscious perception and memory formation). Reprinted with permission.

GWT suggests that a bidirectional broadcast (ignition) corresponds to conscious experience (Dehaene and Changeux, 2011). Figure 1 shows how ignitions in the cortex may emerge from several different regions of interest.

Figure 1 illustrates three different and separate conscious events. One of them is the sensory perception of a single star on a dark night, which plausibly emerges in area V1. The second is the visual experience of a coffee cup in nearby space, which plausibly emerges in the parietal cortex in combination with lower and higher regions of vision. The third conscious event may be a subtle Feeling of Knowing (FOK), as in the tip-of-the-tongue state, which is a semantic and linguistic event, in addition to being closely involved with conscious perception.

Often, multiple gestalts emerge in the same conscious experience.

There are ongoing debates about the role of prefrontal and posterior regions of the cortex in conscious events, but this dichotomy is not obvious, given the interactivity of the cerebral cortex as a whole. It is far more likely that many different cerebral regions interact with each other from moment to moment, and that a static “front vs. rear” contest is simply misleading.

Raccah et al. (2021) make an incorrect claim about Global Workspace Theory (GWT). Raccah et al. themselves cite evidence that supports the role of PfC in a wide range of conscious experiences, including sensory experiences (sometimes called “qualia”). These varieties of conscious experiences involve PfC and other regions (Dehaene et al., 1998). Raccah et al. (2021), in fact, provide a wealth of evidence for GWT-GWD; their hypothesis is contradicted by the very evidence they present.

### Cortex as a Unified Oscillatory Machine

Because almost all neural links in the cortico-thalamic (C-T) system are bidirectional, reentrant signaling between receivers and broadcasting sources may quickly establish task-specific signaling activity. By analogy, a fire department might locate the source of a community-wide alarm and then communicate in a much more task-specific way. Theoretical considerations are important, but a wave of new evidence shows that PfC is directly involved in sensory consciousness, including vision.

We now have direct neuronal studies that show that prefrontal and other cortical regions constantly interact with each other. For example, there is a direct causal link between FEF and early visual processing. Other varieties of consciousness also appear upon direct stimulation of PfC, as shown by iES (Fox et al., 2020; Raccah et al., 2021).

Multiple studies in both humans and macaques show that the prefrontal cortex may be necessary for visual consciousness. Single units in the frontal eye fields (FEF), begin to fire as soon as ~50–60 ms post-stimulus onset. Since visual consciousness emerges after ~300 ms, this finding suggests that the prefrontal cortex activates ultra-rapidly, and suggests that multiple regions of cortex must be involved in visual consciousness. FEF is a region of the prefrontal cortex.

FEF is involved in the eye tremor of physiological nystagmus, which maintains visual stimuli in consciousness. When the constant rapid eye tremor is lost, visual objects are lost from consciousness (see also Kirchner et al., 2009). Therefore, state-of-the-art evidence supports a role for PfC in many conscious functions.

As Libedinsky and Livingstone (2011) write:

“We found that fast responses in FEF strongly correlated with the perceptual report of the animal. It is unlikely that short-latency perceptually correlated activity is inherited from early visual areas, since response latencies in FEF are shorter than those of visual areas with perceptually correlated activity. These results suggest that frontal brain areas are involved in generating the contents of visual perception.”

These authors found FEF activity ~60 ms or less post-stimulus, while visually conscious stimuli are typically found more than ~300 ms post-onset. Thus FEF, a prefrontal region, is involved in pre-conscious visual input processing, contrary to the Raccah et al. (2021) hypothesis.

Kirchner et al. (2009) report similar results, and write that:

“Although the frontal lobes in humans are generally viewed as being involved in high-level cognitive processes, these results indicate that the human FEF is a remarkably quickly activated multimodal region that belongs to a network of low-level neocortical sensory areas.”

Broadly speaking, numerous regions involve both conscious and unconscious processes.

### Cortical Neuropercolation (CNP)

Mathematically, the dynamics of local-global effects have been described by Neuropercolation Theory (Perlovsky and Kozma, 2007; Kozma and Puljic, 2015). Neuropercolation describes cortical phase transitions in the neuropil (layer one). These phase transitions take place between a basal state consisting of competing, local, fragmented components, and a state of high coherence across the hemisphere globally, when the roles of space and spatial differences cease (Kozma and Freeman, 2017). The transition from fragmented states to global coherence appears to be ignited in areas IT/MTL, in the case of visual consciousness (Dehaene and Changeux, 2011).

We believe that it is not fruitful to pose the scientific issues as a contest between PfC and the rest of the cortex. The highly connected, recurrent activations of cortex at multiple levels makes the Raccah et al. claim unlikely.

### Conscious Events may Combine Multiple Gestalt Layers in the Same Experience

Many conscious events are both sensory and semantic. For example, a word in this sentence has sensory properties, but it carries meaning in precisely the same event. Words that are effortful to read and understand also activate the prefrontal and anterior cingulate cortex. Words that involve visual motor actions like playing tennis, also recruit motor regions of the cortex.

It is therefore misleading to make gross anatomical distinctions between prefrontal and other cortical regions.

### Conscious Events Go Beyond the Senses

Some authors seem to restrict the term “consciousness” to sensory events (Raccah et al., 2021). This is an overly narrow definition that excludes numerous conscious experiences, such as feelings of effort, interoceptive emotional feelings, conscious beliefs and ideas, endogenous visual imagery, and inner speech.^1^

In science, the way we ask our questions is crucial, because our questions are likely to bias our answers.

In short, the work by Raccah et al. is a worthy effort to make a systematic study of the role of various cortical regions in consciousness. Obviously, the authors may not claim that they provide a final word in this field. We feel a bit more caution would be in order to avoid making overly ambitious statements, which would inevitably prove wrong or fade into obsolescence as research progresses. A number of workers in the field emphasize the deep and rapid interactivity of numerous regions and connectivities in the C-T core. That dynamical view of the C-T core should give us pause about proposing dichotomies that may not be in evidence.

In summary, GWT-GWD does not deny the role of local regions, rather it integrates them by way of a unifying theory (Baars et al., 2013; Baars and Geld, 2019).

### Metacognition: Using the Prefrontal Cortex to Think About Thinking

The GW concept, in itself, enables metacognitive processes from unconscious onlooking systems viewing the conscious flow in the global workspace. GWT suggests the plausibility of both conscious and unconscious metacognition. These should be considered separately.

In particular, we make a fundamental distinction between:

a. CONSCIOUS metacognition, in which humans consciously think about their own conscious experiences—editing a text like this is one obvious example.
b. UN-conscious metacognition, where UN-conscious specialized processors monitor the conscious global workspace to interrupt the conscious flow, or otherwise intervene if the UN-conscious “critic” detects a serious problem.

This is one basic advantage of the GW architectures first developed by Allen Newell and his team. Stan Franklin and many other computer scientists and AI researchers have long exploited this feature of GW architectures. In neural net mathematics a similar strategy is used in so-called “critic neural networks” (Si, 2005).

We believe that human conscious cognition inherently allows for both conscious thought about the conscious stream, as well as UN-conscious comments on the conscious stream.

For example, it would be easy to have an unconscious snake detector as one of the many unconscious specialists watching the brain equivalent of a global workspace. Such a snake detector may exist in the two amygdalae near the tip of each temporal lobe. If we were conscious of a complex image of a bush in the hot African sun, the unconscious snake circuit would identify the real snake and separate it from the shadow of a branch. Snakes use camouflage, of course, and our brains do have specialized snake circuits. Unconscious detection of snake-branch ambiguities in the conscious stream would be highly adaptive.

There is a great deal of evidence that all parts of the cortex are involved with a variety of conscious experiences. The cortex is deeply engaged with every kind of conscious experience event: sensory consciousness (including so-called “qualia”); endogenous sensory and non-sensory events such as visual imagery, inner speech, conscious beliefs, conscious decisions, consciously experienced emotions, and feelings of knowing (FOKs). The traditional view of the prefrontal cortex is that it includes self reflection and metacognition (Qiu et al., 2018).

Metacognition has also been claimed to be a distinctive requirement for consciousness, but this claim has pros and cons (Shea and Frith, 2019). Simply reporting one's fleeting perceptual content involves one kind of metacognition, since behavioral report is about the preceding sensory stimulus. Conscious cues often trigger subsequent processing, such as mental rehearsal for strengthening recall, and that task also refers to the first conscious exposure. Thus metacognition is extremely common in the stream of consciousness (Yeung and Summerfield, 2012).

Franklin's LIDA set of simulations of Global Workspace Theory employs metacognition for numerous tasks, which is relatively easy to program, since any “globally broadcast” event can also trigger metacognitive agents (Franklin et al., 2012).

Yet there are conscious experiences that minimize metacognition, namely absorbed experiences, as in the case of “flow” experiences and any other total involvement with a dense flow of events. After an absorbed state, it is generally difficult for people to remember metacognitive judgments from the absorbed state (Csikszentmihalyi and Lebuda, 2017). It seems as if “deep absorption” minimizes the capacity for conscious metacognition, which makes sense in light of the limited capacity constraint. Absorption may drive out conscious metacognition. It is hard to reflect about inaccessible contents that occurred during absorption. However, during absorption it is entirely possible that unconscious metacognition continues, which would be suggested by our ability during absorption to be interrupted by an unexpected fire alarm, presumably detected unconsciously.

As for metacognitive experiences, they are so common in the ordinary stream of consciousness that they must also involve the broad cerebral cortex (Baars and Geld, 2019).

## Conclusion: The Dynamical Connectome of Cortex

A wide range of experimental and theoretical studies in the field of consciousness emphasize the complex and rapid interactivity of numerous regions and connectivities in the C–T core. That dynamical view of the C–T core should give us pause about proposing dichotomies that may not be in evidence.

From a theoretical viewpoint, Baars et al. (2013) suggest that the cortico-thalamic system is inherently dynamic, a view also taken by Pribram (1991), Edelman and Tononi (2000), Freeman et al. (2008), Dehaene and Changeux (2011), Edelman et al. (2011), and others. If we view the epicenter of conscious events in cortex to be dynamic rather than anatomically static, the question of prefrontal involvement becomes more nuanced. Most recently, Deco et al. (2019) define a functional “rich club” of active cerebral nodes and connectivities that may function as a dynamic global workspace, one that is not rigidly tied to a single anatomical region of cortex. There may be other ways to identify global workspace dynamics, but this appears to be a well-specified candidate.

Consciousness studies have been undergoing rapid development, in part, due to new experimental techniques and brain monitoring. The GW “family” of theories has been at the forefront of these developments, and continues to make novel predictions. Relevant mathematical advances are also emerging, including spatio-temporal dynamic network and graph theory approaches. We hope that these points will help to clarify the evidence about GWT and the conscious brain.

## Author Contributions

All authors listed have made a substantial, direct and intellectual contribution to the work, and approved it for publication.

## Funding

This work was supported by the Center for the Future Mind, Florida Atlantic University (to BB).

## Conflict of Interest

NG is founder of MedNeuro, Inc. The remaining authors declare that the research was conducted in the absence of any commercial or financial relationships that could be construed as a potential conflict of interest.

## Publisher's Note

All claims expressed in this article are solely those of the authors and do not necessarily represent those of their affiliated organizations, or those of the publisher, the editors and the reviewers. Any product that may be evaluated in this article, or claim that may be made by its manufacturer, is not guaranteed or endorsed by the publisher.

## Footnotes

^1^When we talk about thinking we often use the word “explicit” rather than “conscious” and it is a plausible source of confusion in the literature. To make our views abundantly clear, therefore, when we use the word conscious, we mean to include both the terms “explicit cognition” as well as “conscious perception.” Clarifying these terms relates directly to understanding and communicating the evidence. Some authors seem to restrict the term “consciousness” to sensory events (Raccah et al., 2021). This is an overly narrow definition that excludes numerous conscious experiences, such as feelings of effort, interoceptive emotional feelings, conscious beliefs and ideas, endogenous visual imagery, and inner speech. This definitional heterogeneity is made worse by the widespread use in the scientific literature (Frith and Frith, 2008) of terms like “explicit cognition” instead of “conscious cognition.” In everyday English, the term “conscious” extends beyond sensation and perception, and applies to ideas, voluntary decisions, memory recall, etc., functions that have long been thought to involve the PfC. For all intents and purposes, the term “explicit” means “conscious” or “consciously mediated.” This is still a serious source of confusion today. There is no question that the prefrontal cortex is intimately associated with explicit mental functions — that is to say, those conscious events that can be explicated. Perceptual contents are not explicit in this sense, because people in general cannot explicate the visual sense, for example: the terms “conscious” and “explicit” are often confounded. They are closely related, but not identical in meaning. Thus, a great many definitional problems result from a confusion of labels.
